# Silver ions blocking crystallization of guanosine-based hydrogel for potential antimicrobial applications†

**DOI:** 10.1039/c8ra02500b

**Published:** 2018-04-27

**Authors:** Hui Feng, Yuqi Du, Fan Tang, Ning Ji, Xuefeng Zhao, Hang Zhao, Qianming Chen

**Affiliations:** State Key Laboratory of Oral Diseases, West China Hospital of Stomatology, Sichuan University Chengdu Sichuan 610065610041 P. R. China zhao.zxf@gmail.com zhaohangahy@scu.edu.cn; XiangYa Stomatological Hospital, Central South University Changsha Hunan 410000 P. R. China

## Abstract

In this work, the detailed crystallization process of 2′-deoxy-2′-fluoroguanosine (^F^G_d_) hydrogel has been studied using single crystal X-ray diffraction, variable-temperature nuclear magnetic resonance (VT-NMR), and scanning electron microscopy (SEM). Both solid and solution results indicated that the K^+^-mediated G-quartet structures were unstable and easily resulted in the breakdown of the hydrogel to form linear ribbon structures by forming mimic reverse Watson–Crick base pairs between the two faces with an intermolecular hydrogen-bond (N10H–O11). Accordingly, Ag^+^ was introduced to block the crystallization of ^F^G_d_ to form long lifetime stable supramolecular hydrogel (>6 months) and possible silver-ions-mediated base pair motifs were suggested *via* NMR, UV, and mass spectroscopy (MS) in combination with powder X-ray diffraction (PXRD) and circular dichroism spectroscopy (CD). Furthermore, ^F^G_d_Ag hydrogel exhibited low toxicity for normal oral keratinocyte cells (NOK-SI) and good antibacterial activities for *Fusobacterium nucleatum in vitro*.

## Abstract 2

Ag^+^ was introduced to block the crystallization of ^F^G_d_ forming long lifetime stability supramolecular hydrogel (>6 months), which displayed low toxicity for NOK-SI cell and good antibacterial activities.

## Graphical abstract

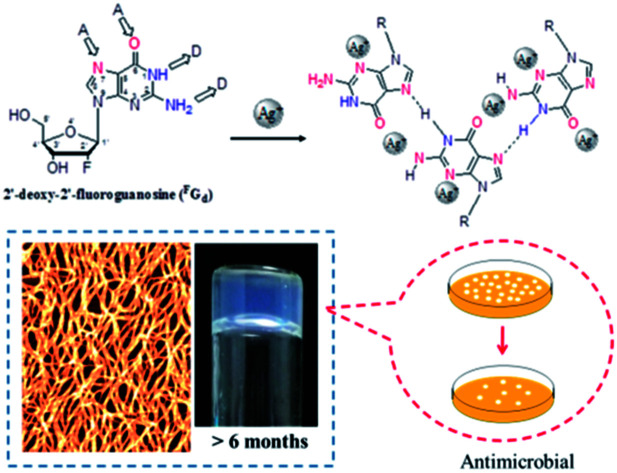

## Introduction

Supramolecular hydrogel self-assembled from low-molecular-weight gelators have gathered increasing attention over the past few decades owing to their potential applications in various fields including tissue scaffolding, drug delivery system, and functional biomaterials.^1–13^ Among the five natural nucleosides, guanosine and its derivatives are of special interest as their self-complementary hydrogen-bond edges and aromatic surfaces, which promote them to further self-associate into highly ordered structures such as dimers, ribbons, and macrocyclic tetramers and even form three-dimensional (3D) gels containing certain alkali metal ions in aqueous or organic media.^14–19^ Although there has been a long history of guanosine-derivative-based hydrogels since Bang demonstrated the formation of gels with guanylic acid in 1910,^19–24^ a major limitation of guanosine-based hydrogels was their poor lifetime stability owing to their crystallization within hours at room temperature, which hinders their latent applications in biological or pharmaceutical fields.^24^ To overcome the above limitation, guanosine derivatives,^25–34^ such as 5′-hydrazide-guanosine, 8-methoxy-2′,3′,5′-tri-*O*-acetylguanosine, and guanosine-borate esters, have been developed to improve the lifetime stability of hydrogels.

**Fig. 1:**
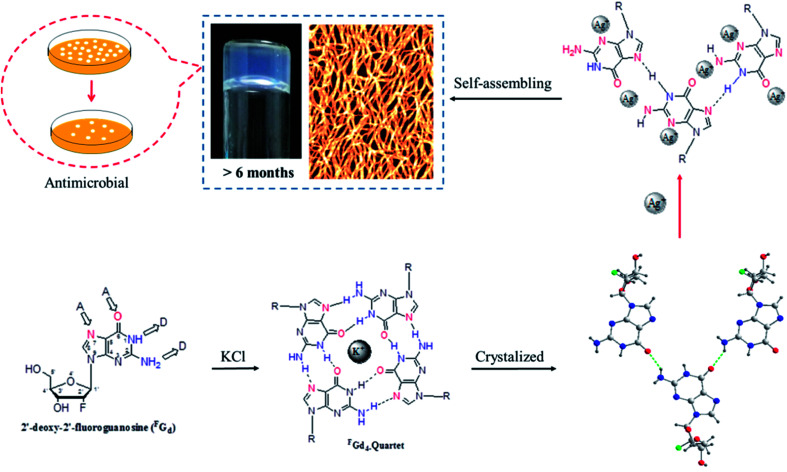
Schematic illustration of a long lifetime stable supramolecular hydrogel formed when silver ions were used to block the crystallization of ^F^G_d_. The hydrogel further showed highly antimicrobial activities.

However, to the best of our knowledge, the crystallization process of guanosine-based hydrogels has not been explored yet. Thus, determining their delicate structural balance between gelation and crystallization is critical, not only to gain more insight into the reasons for crystallization and further understand how to design new guanosine-based gelators but also to tune their lifetime stability and mechanical properties to develop multifunction supramolecular materials in the future. Inspired by this, the crystallization process of 2′-deoxy-2′-fluoroguanosine (^F^G_d_) hydrogel has been studied in this work. ^F^G_d_ has the same base and similar sugar moiety as guanosine. It was first synthesized by J. Imura in 1981 and thereafter, it was observed to exhibit significant anti-virus activities for influenza A and B, herpes simplex virus (HSV), and H7N1.^35–39^ Furthermore, Christopher J. L. suggested that ^F^G_d_ is a powerful anti-favoring tool to manipulate G-quadruplex polymorphism and folding topology.^40^ Recently, the gel properties of ^F^G_d_ have been investigated by our group and the results indicated that ^F^G_d_ forms a transparent hydrogel only in the presence of K^+^.^41^ In this work, the detailed crystallization process of ^F^G_d_ hydrogel was first studied using single crystal X-ray diffraction, VT NMR, SEM, PXRD, and CD. Based on the above information, we observed that silver ions could not only block the crystallization of ^F^G_d_ but also induce ^F^G_d_ to form a long-term stable supramolecular hydrogel with favorable antimicrobial activities (Fig. 1).

## Result and discussion

**Fig. 2:**
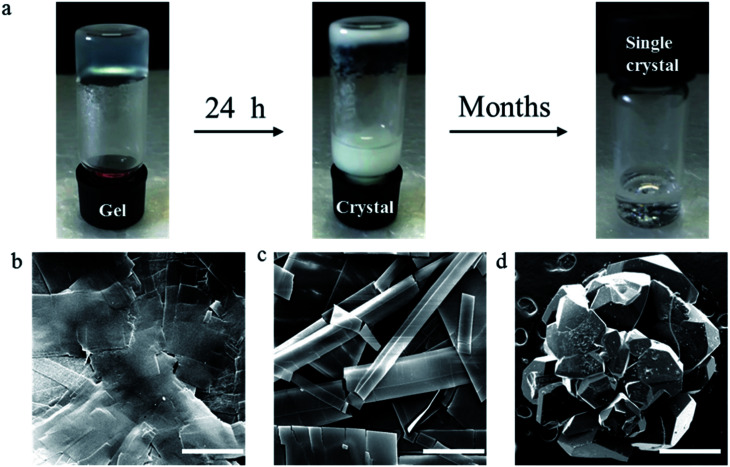
(a) Images of the crystal process of ^F^G_d_ hydrogel at a concentration of 1.4 mg per 100 μL of 0.2 M KCl solutions. (b–d) SEM images of the xerogels, crystal, and single crystal prepared from ^F^G_d_. Scale bars: 10 μm (b), 25 μm (c), and 0.5 mm (d).

Recently, we reported the gel properties of ^F^G_d_ and the results indicated that ^F^G_d_ forms a transparent gel in the presence of K^+^ and crystallizes in the presence of Li^+^, Na^+^, Rb^+^, and Cs^+^.^41^ The possible self-assembling process of ^F^G_d_ hydrogel is shown in Fig. S1a† according to the previous guanosine-based hydrogel. To obtain an insight into the microstructure of the hydrogel formed by ^F^G_d_, SEM was performed on xerogels containing KCl at a lower concentration to avoid the negative impact caused by the crystallization of salt. 3D networks with porous-like structures consisting of intensive slices were observed and the diameter of the hole was approximately 150 μm (see ESI, Fig. S1b and c†). Accordingly, atomic force microscope (AFM) measurements were carried out and a similar slice structure was obtained. Notably, some flexible fibers with a diameter of about 10–20 nm were observed on the surface of slices. These fibers inter-tangled with each other to form highly ordered slice structures and thereafter formed 3D hydrogel networks. Nevertheless, ^F^G_d_ hydrogel was easily crystallized within hours similar to other guanosine-based hydrogels. Recent studies gave much evidence to explore the properties of gel to crystal transition for peptide, amino acid and sugar derivatives.^42–45^ However, there is no literature to report the detail crystallization process of guanosine-based hydrogels. To find out and understand the reason for the crystallization of ^F^G_d_ hydrogel, the visualize dynamic crystal process of ^F^G_d_ hydrogel has been investigated as shown in Fig. 2a. First, ^F^G_d_ formed a transparent gel at the concentration of 1.4 mg per 100 μL of 0.2 M KCl solution. Subsequently, the nucleoside ^F^G_d_ began to crystallize and the hydrogel broke down after 24 h. Eventually, we got the single crystal of ^F^G_d_ in the above solution after several months, which was, to the best of our knowledge, the first free guanosine-based nucleoside crystal formed from hydrogel and would provide us more detailed information about the reason for crystallization at molecular level. Meanwhile, the crystal process was also monitored by SEM at micro level (Fig. 2b). The results indicated that the micron-scale slice-like structures gradually grew to piece-like and even colorless millimeter-scale granule-like structures while the gel transformed to a single crystal.

**Fig. 3:**
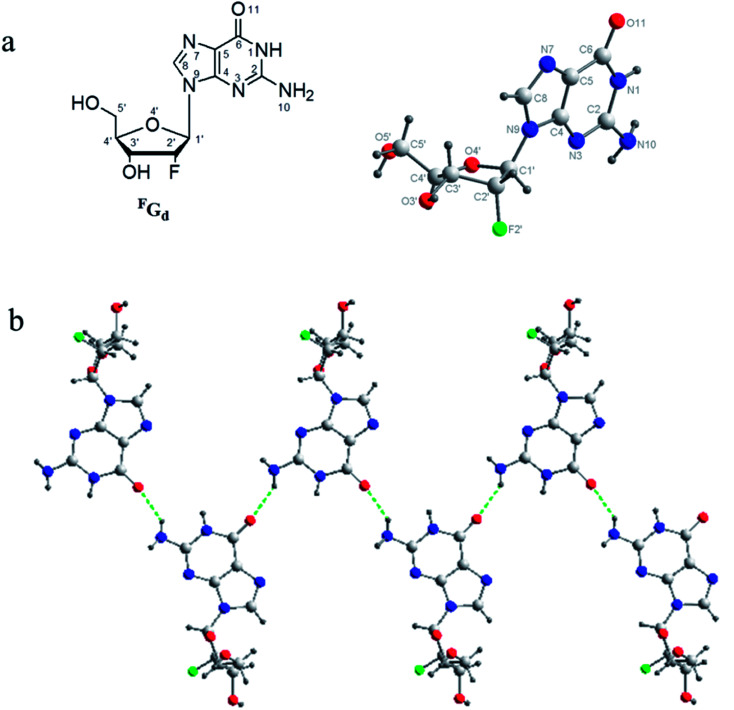
(a) Molecular structure with systematic numbering and single crystal structure of ^F^G_d_, which adopted an *anti*-conformation with an N-type (3′-*endo*) sugar puckering and 5′-OH at ap position. (b) A detailed view of the mimic reverses Watson–Crick base pairs in the solid state of ^F^G_d_ and the repeated hydrogen bonds unit in the entire assembly. Atoms were coded as follows: red, oxygen; blue, nitrogen; gray, carbon; green, fluorine; black, hydrogen.

Furthermore, to get more detail information about the crystal from the hydrogel in molecular level, the single crystal structure of ^F^G_d_ has been analyzed carefully from three levels: monomer molecular, base pair motif and three-dimension structure. Firstly, the monomer molecular structure with systematic numbering and the single crystal structure of ^F^G_d_ were shown in Fig. 3. The *anti*/*syn* conformation of the glycosyl bond was paramount to the canonical purine or pyrimidine nucleosides, which could be defined by the torsion angle *χ* (O4′–C1′–N9–C4). The torsion angles *χ* of ^F^G_d_ were measured to be −93.15(33)°, indicating that it adopted a normal *anti* conformation. Sugar puckering is another significant conformational parameter defined by the pseudorotation phase angle (*P*) and maximum puckering amplitude (*τ*_m_). Two ranges of pseudorotation phase angle were initially observed in natural and synthetic nucleosides: C3′-*endo* with 0° ≤ *P* ≤ 36° (North) or C2′-*endo* with 144° ≤ *P* ≤ 180° (South). ^F^G_d_ adopted a typical N-type conformation with a twist of C3′-*endo* (^3^T_2_, N, *P* = 15.6(4)°, *τ*_m_ = 33.6(7)°). Moreover, it was also a crucial structural parameter for the orientation of the 5′-hydroxyl group defined by the torsion angle *γ* (O5′–C5′–C4′–O4′) relative to the sugar ring. For ^F^G_d_, it was in the ap (*gauche*, *trans*) range with the O5B′–OH at the axial position and pointing outside the sugar ring (*γ* = 61.98(33)°). Secondly, as ^F^G_d_ has the same base moiety as guanosine, which has three faces with hydrogen bonding donors and acceptors, it can self-assemble into complex and unique supramolecular structures, such as dimers, ribbons, and tetramers. In this case, the aforementioned structures were not obtained as the hydrogels broke down and it was only found that ^F^G_d_ could form mimic reverse Watson–Crick base pairs between two faces with an intermolecular hydrogen-bond (N10H–O11), which extended to form a linear ribbon structure with sugar residues located on both sides.

**Fig. 4:**
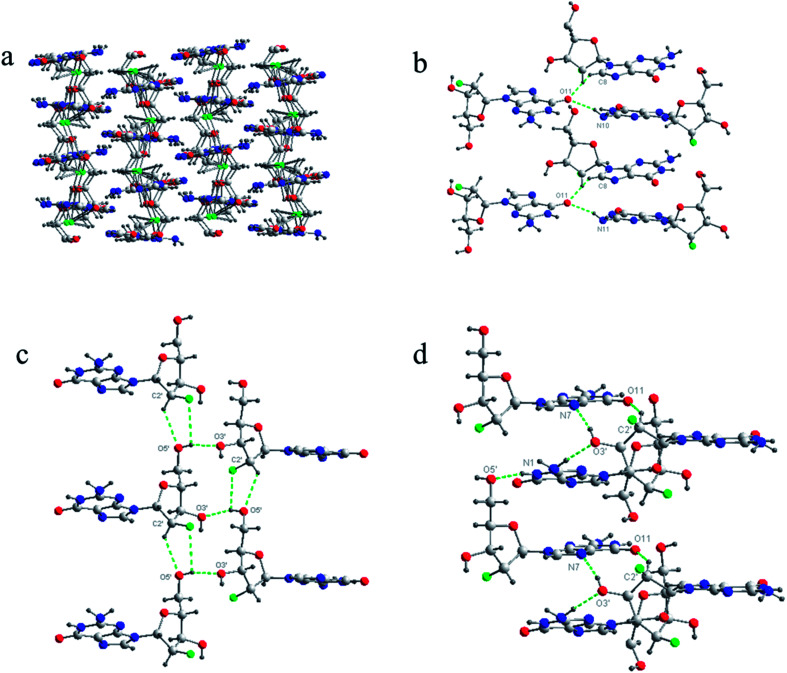
Complicated hydrogen bond networks of ^F^G_d_. (a) The overall multilayered supramolecular structure of ^F^G_d_. (b) The interactions between bases. (c) The interactions between sugars. (d) The interactions between base and sugar. Nine hydrogen bond units repeated across the different layers in the entire assembly were highlighted in green color. Atoms were coded as follows: red, oxygen; blue, nitrogen; gray, carbon; green, fluorine; black, hydrogen.

Finally, based on the base pair motif, a multilayered structure was formed at the 3D supramolecular level with complicated hydrogen bond networks including additional contributions from sugar residues (Fig. 4). To clearly display the hydrogen bond networks, the 3D supramolecular network was divided into three aspects: base–base, sugar–sugar, and base–sugar interactions of different layers. The interaction between base moieties is shown in Fig. 4b. O11 held three molecules from three neighboring layers together as a bridge with two hydrogen bonds, and it was connected to C8H of one molecule and N10H of another molecule. The sugar–sugar interactions are displayed in Fig. 4c. The C2′–H of one molecule was directly connected to the O5′ of another molecule. 5′-OH linked together three molecules from three adjacent layers with two hydrogen bonds (2′F–5′OH–3′O). The interactions of base–sugar are shown in Fig. 4d. There were four hydrogen bonds between four molecules at three neighboring layers (5′O–N1H, N7–3′OH, 3′O–N10H, O11–C2′H). In general, a complicated hydrogen bond network formed with nine hydrogen bonds was repeated infinitely throughout the assembly process of the 3D multilayered supramolecular structure in the single-crystal state.

In order to explore their self-assembling properties in solution state, VT NMR spectrometry was used to investigate the formation of hydrogen bonds of ^F^G_d_. As shown in Fig. S2,† the chemical shift of N10H atom of the amino group moved from *δ* 6.60 (298 K) to *δ* 6.46 ppm (338 K) with Δ*δ* = 0.14 ppm, the chemical shift of 3′-OH atom of the hydroxyl group moved from *δ* 5.69 (298 K) to *δ* 5.53 ppm (338 K) with Δ*δ* = 0.16 ppm, and the chemical shift of 5′-OH atom of the hydroxyl group moved from *δ* 5.15 (298 K) to *δ* 4.97 ppm (338 K) with Δ*δ* = 0.18 ppm. These results indicated that these hydrogen atoms participated in the formation of intermolecular hydrogen bonds. Another hydrogen atom (N1H) from the imino group at *δ* 10.73 ppm was assigned as the intramolecular hydrogen bond as its chemical shift remained unchanged upon the increase in the temperature. To this end, ^1^H–^1^H nuclear overhauser effect (NOE) experiments was performed and they demonstrated that the C_8_–H⋯NH_2_ and NH NOE signals (illustrated by the empty boxes, see ESI, Fig. S3†) were not observed, which was consistent with the solid state.

**Fig. 5:**
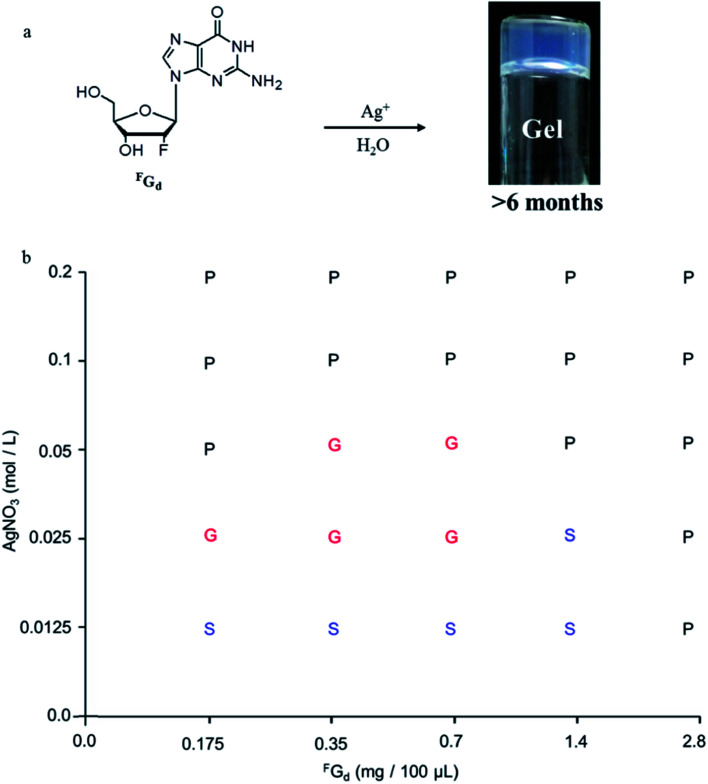
Preparation and characterization of ^F^G_d_Ag hydrogel in water. (a) The formation of a long-lasting stable supramolecular hydrogel *via* Ag-ions-mediated ^F^G_d_ in water at room temperature. (b) Phase diagram of gelation of ^F^G_d_ at different concentrations of aqueous AgNO_3_. G: gel, S: solution, P: precipitate.

Both solid and solution results indicated that the K^+^-introduced G-quartet structure was not stable and easily broke down in hydrogel and subsequently formed a linear ribbon structure with mimic reverse Watson–Crick base pairs between the two faces with an intermolecular hydrogen-bond (N10H–O11), which may be the reason for the propensity of guanosine-based hydrogel to crystallize over time. To block the crystallization of ^F^G_d_ hydrogel getting a long lifetime stable supramolecular hydrogel, introducing stronger metal bonds would be a good choice. Previously, researchers determined that metal ions such as Pt^2+^, Hg^2+^, and Ag^+^ tended to alter hydrogen bonding patterns between guanosine molecules by the coordination of the electron-rich nitrogen and oxygen groups around the purine ring to produce a range of metal ion-linked H-bonded architectures.^34,46,47^ For example, Kraatz *et al.* reported that an Ag^+^ induced G gel was exploited for the light triggered *in situ* fabrication of uniform AgNPs within a gel to make a nano–bio hybrid material; Mann *et al.* found that supramolecular hydrogels produced by spontaneous self association of disodium guanosine 5′-monophosphate (Na_2_5′GMP) in the presence of Ag^+^ ions. Inspired by this, Ag^+^ was introduced in this work and the results showed that ^F^G_d_ can not only form hydrogel in the presence of silver ions but also demonstrate long lifetime stability (>6 months). Furthermore, ^F^G_d_ hydrogelation experiments were carried out at different concentrations of AgNO_3_ and ^F^G_d_ (0.0125 M to 0.2 M AgNO_3_ and 0.175 mg to 2.8 mg of ^F^G_d_ per 100 μL of solution). The phase diagram in Fig. 5 revealed that the formative qualification of the hydrogel. The concentration control between AgNO_3_ and ^F^G_d_ was observed to play a critical role in the formation process of ^F^G_d_ hydrogel. Interestingly, ^F^G_d_ tended to form a hydrogel at low silver ion concentration, which suggested that the hydrogel had significant potential for biological or medical applications.

**Fig. 6:**
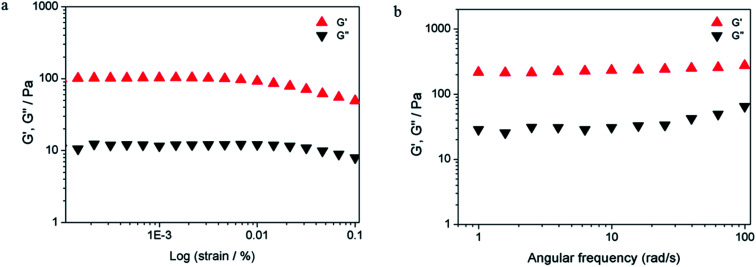
Evaluation of *G*′ and *G*′′ as a function of the strain (a) and frequency sweep (b). The compound was measured at the concentration of 0.7 mg per 100 μL in 0.025 M AgNO_3_ solutions at 25 °C.

Furthermore, the mechanical properties of the hydrogel were analyzed in detail using rheological measurements. The storage modulus (measurement of elastic property) and loss modulus (measurement of fluidity) were expressed as *G*′ and *G*′′, respectively. As shown in Fig. 6, the typical elastic nature of ^F^G_d_ + Ag^+^ (^F^G_d_Ag) hydrogel was evident from the fact that *G*′ > *G*′′ (solid-like behavior) *via* strain amplitudes ranging from 0.001 to 0.1% at 6.28 rad s^−1^. Moreover, the frequency sweep experiments of ^F^G_d_Ag hydrogel were performed in the angular frequency range of 0.1–100 rad s^−1^ under an initial strain of 0.1% and the results showed that ^F^G_d_Ag hydrogel had a higher storage modulus *G*′ than loss modulus *G*′′ over the entire applied frequency range, indicating that it exhibited a solid-like behavior.

**Fig. 7:**
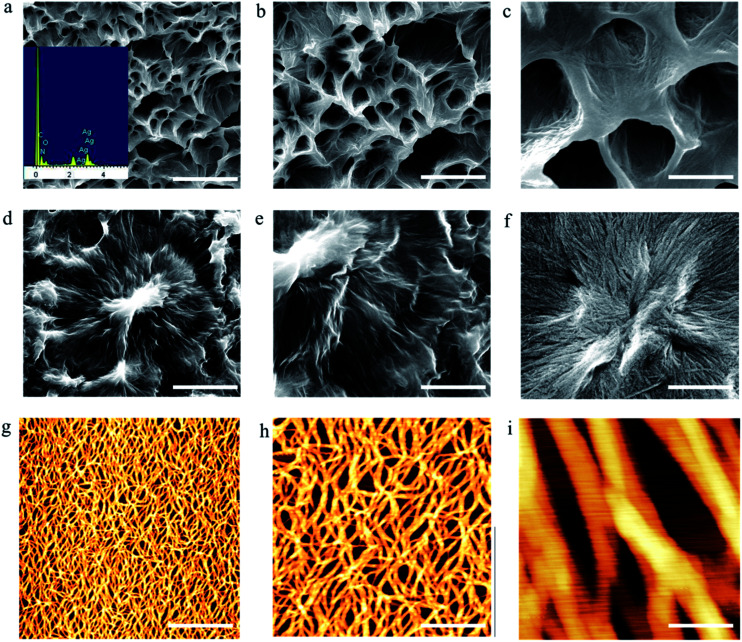
Microstructures of ^F^G_d_ hydrogel at the concentration of 0.7% in 0.025 M AgNO_3_ solution. (a–f) SEM images of ^F^G_d_ hydrogel. (g–i) AFM images of ^F^G_d_ hydrogel. Scale bars: 5 μm (a and d), 1.5 μm (b and e), 500 nm (c and f), 1.5 μm (g), 500 nm (h), and 50 nm (i).

To obtain microstructures of the ^F^G_d_Ag hydrogel, we prepared xerogels for SEM observations (Fig. 7a–f). The hydrogels were prepared in a sample tube (0.7 w/v%) containing 0.025 M AgNO_3_ and they were subsequently frozen. The frozen samples were lyophilized using a vacuum pump. Prior to examination, the xerogel was attached to the silica wafer and coated with a thin layer of gold. The SEM images revealed the three-dimensional uniform porous-like structures with a diameter of approximately 500 nm. Careful inspection of the images revealed that flexible fibers with a diameter of approximately 20–30 nm and several micrometers in length were formed spontaneously during the formation of hydrogel. The fibers intertangled with each other to form highly ordered film-like structures. Energy-dispersive X-ray spectroscopy analysis confirmed the presence of Ag in the fibers. AFM was carried out to further analyze and evaluate the structures of the hydrogel. Fig. 7g–i reveals the existence of an interconnected fibrous network with a diameter of approximately 20–30 nm, height of 6–8 nm, and length of several micrometers, which are consistent with the above results.

**Fig. 8:**
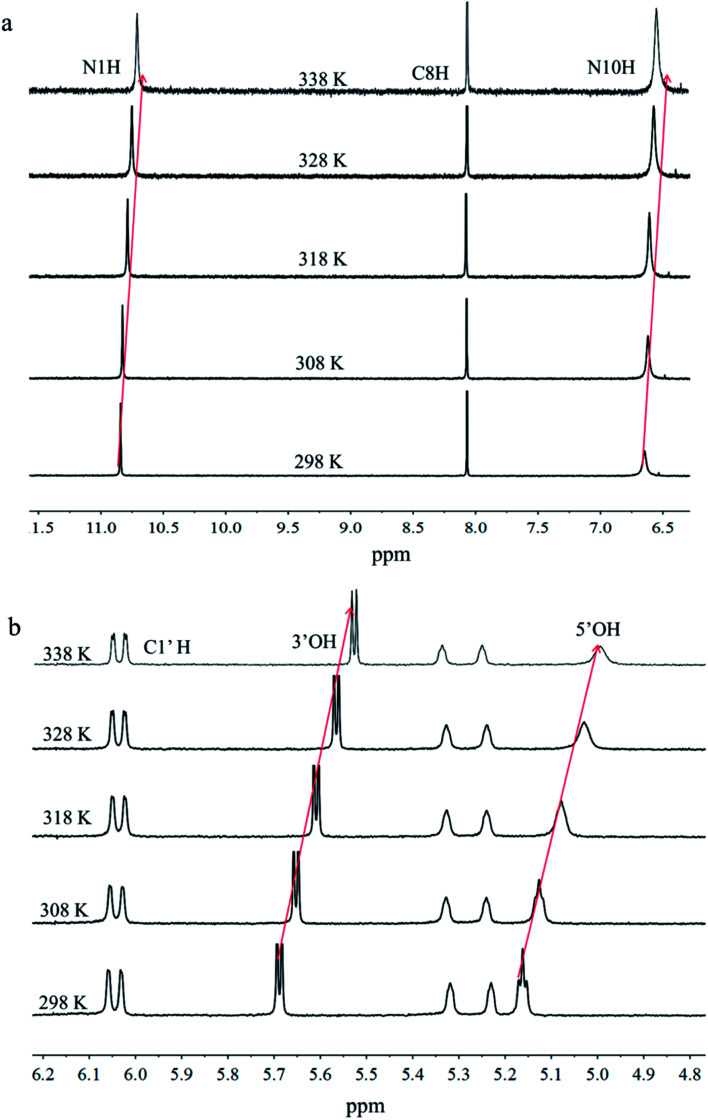
VT ^1^H NMR spectra of ^F^G_d_Ag recorded from 298 K to 338 K.

To explore more molecular-level evidences of the hydrogel, CD spectroscopy, PXRD, and NMR were carried out. Previous reports indicated that a planar quartet system show peaks at 240 nm and 260 nm in CD spectrum.^28^ Here, the ^F^G_d_Ag hydrogel showed two negative peaks at 220 and 290 nm (see ESI, Fig. S4†), which indicated that the G-quartet structures were not observed in this hydrogel. As shown in Fig. S5,† the freeze-dried sample of ^F^G_d_Ag hydrogel was studied using PXRD and it exhibited similar patents for ^F^G_d_Ag hydrogel and the crystal of ^F^G_d_ hydrogel in the presence of K^+^. All of them exhibited a significant peak at 2*θ* ≈ 6.0° and 26.8° (*d* = 14.5 Å and 3.4 Å, respectively) consistent with the monomeric length and Pi–Pi stacking distance between two layers of G rings.^31^ Subsequently, ^F^G_d_ and ^F^G_d_Ag were characterized using ^1^H NMR spectra at 298 K (see ESI, Fig. S6†). The results showed that N10H peak almost disappeared when Ag^+^ was introduced to ^F^G_d_, indicating that the amino group may participate in the silver base pair. VT NMR experiments were carried out to further investigate the formation of hydrogen bonds of ^F^G_d_Ag. As shown in Fig. 8 and Table S1,† the chemical shift of N1H atom was from *δ* 10.84 (298 K) to *δ* 10.64 ppm (338 K) with Δ*δ* = 0.20 ppm, the chemical shift of N10H atom of the amino group was from *δ* 6.65 (298 K) to *δ* 6.47 ppm (338 K) with Δ*δ* = 0.18 ppm, and the peak began to increase in relative intensity when the temperature increased from 298 K to 338 K. The chemical shift of 3′-OH atom of the hydroxyl group moved from *δ* 5.69 (298 K) to *δ* 5.53 ppm (338 K) with Δ*δ* = 0.16 ppm and the chemical shift of 5′-OH atom of the hydroxyl group moved from *δ* 5.16 (298 K) to *δ* 4.98 ppm (338 K) with Δ*δ* = 0.18 ppm. These results demonstrated that these hydrogen atoms participated in the formation of intermolecular hydrogen bonds.

**Fig. 9:**
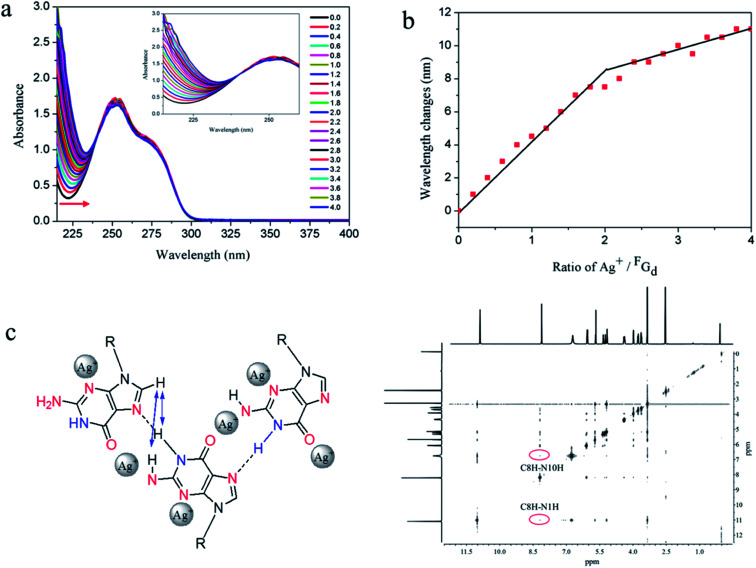
Analysis and evaluation of possible silver-ions-mediated base pair in ^F^G_d_Ag hydrogel. (a) UV spectrophotometric titration of 0.25 μM ^F^G_d_ with the increase in Ag^+^ concentration measured in H_2_O. (b) Graphs of the ratio of silver ion/duplex *versus* absorbance changes measured at different wavelengths. (c) The proposed silver-mediated self-assembling structure of ^F^G_d_ and its ^1^H–^1^H NOE spectra.

Stoichiometric titration experiments were performed to verify the formation of silver-mediated pairs and determine the amount of silver ions bound to the nucleoside ^F^G_d_. As shown in Fig. 9, the changes in UV wavelengths were plotted *versus* the ratio of silver equivalents/^F^G_d_, suggesting that two silver ions were captured by one ^F^G_d_ (Fig. 9a and b). Accordingly, positive mode electrospray ionization-mass spectroscopy analysis was carried out and the corresponding silver complexes of 1 : 1 and 1 : 2 peaks at 475.1 and 624.1, respectively, were observed (see ESI, Fig. S7†), which provided powerful evidence further indicating that the two silver ions were captured by one ^F^G_d_. Based on the above results, the possible silver-mediated base pair motifs are shown in Fig. 9c. To validate the assumption, ^1^H–^1^H NOE experiments were carried out and the results suggested that C8–H produced a strong NOE at NH2a, NH2b, and NH simultaneously (Fig. 9c), which provided an unambiguous interpretation of the G-ribbon structures.

**Fig. 10:**
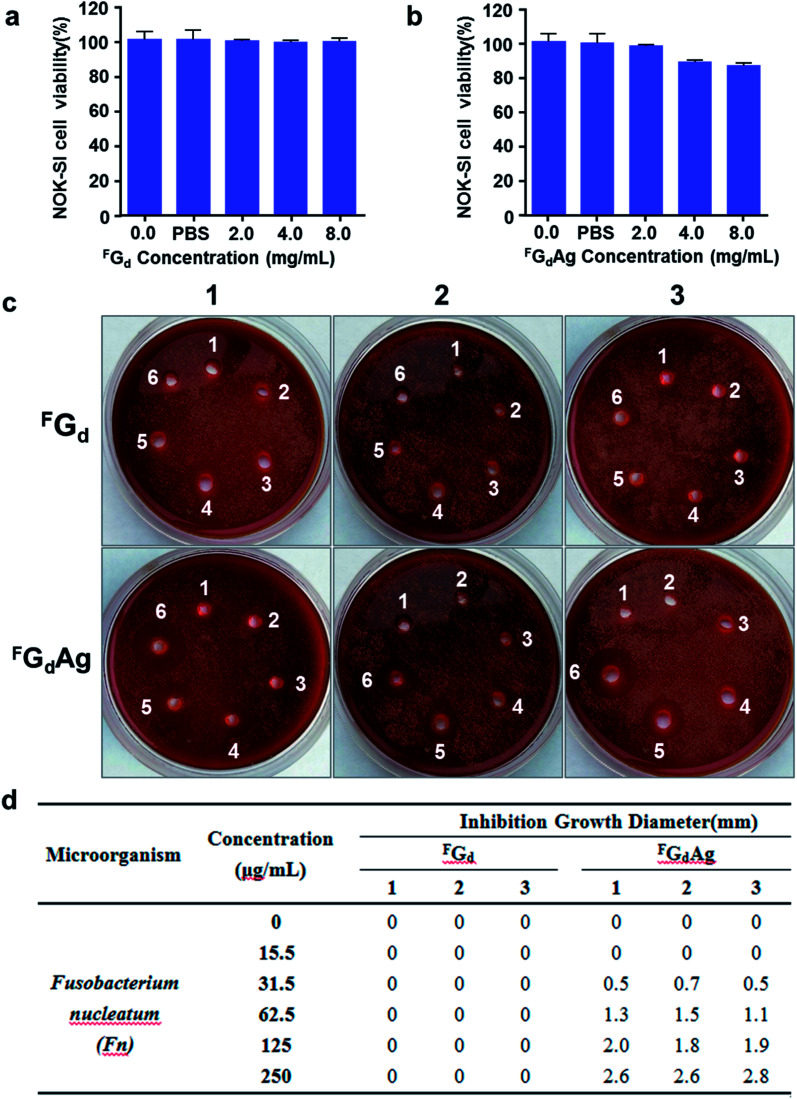
Demonstration of low cytotoxicities and excellent antimicrobial efficiencies of ^F^G_d_ and ^F^G_d_Ag *in vitro*. (a and b) CCK8 assay of the cytotoxicities of ^F^G_d_ and ^F^G_d_Ag on immortalized normal oral keratinocyte cells (NOK-SI). The results represent mean ± SD. (c) The antibacterial activities *in vitro via* evaluating the inhibition growth diameter of ^F^G_d_ and ^F^G_d_Ag hydrogels against *Fusobacterium nucleatum*. In the sterilization experiment, (d) the concentrations of ^F^G_d_ and ^F^G_d_Ag hydrogels were 0 μg ml^−1^ (1); 15.5 μg ml^−1^ (2); 31.5 μg ml^−1^ (3); 62.5 μg ml^−1^ (4); 125 μg ml^−1^ (5) and 250 μg ml^−1^(6), respectively. The dates represent the growth inhibition diameter (in mm) of each sample in three independent experiments.

*Fusobacterium nucleatum* and *Porphyromonas gingivalis*, two Gram-negative anaerobes, are the most abundant microorganisms present in the oral cavity during periodontal disease.^48,49^ In this study, the antibacterial activities of ^F^G_d_ and ^F^G_d_Ag hydrogels *in vitro* were preliminarily evaluated. Initially, we assessed the biocompatibility of ^F^G_d_ (^F^G_d_ dissolved in PBS) and ^F^G_d_Ag hydrogels *in vitro*, the cell viability of the immortalized normal oral keratinocyte cells (NOK-SI) was assessed using CCK8 assay. As shown in Fig. 10, when the concentration of ^F^G_d_ and ^F^G_d_Ag increased to 2.0 mg mL^−1^, the viability of the NOK-SI cell line was higher than 97%. As the concentration of ^F^G_d_ and ^F^G_d_Ag increased to 8.0 mg mL^−1^, the viability of NOK-SI cell line was higher than 86%. As expected, these results indicated that the ^F^G_d_ and ^F^G_d_Ag hydrogels had only slight toxicity *in vitro* to be used as a safe biomaterial. Furthermore, the preliminary *in vitro* antimicrobial activities of ^F^G_d_ and ^F^G_d_Ag hydrogels on *Fusobacterium nucleatum* and *Porphyromonas gingivalis* were evaluated by measuring the inhibition growth diameter and minimal bactericidal concentrations (MBCs). The results presented ^F^G_d_ and ^F^G_d_Ag hydrogels had no obvious inhibition effect on *Porphyromonas gingivalis.* However, ^F^G_d_Ag hydrogel exhibited excellent antibacterial activities for *Fusobacterium nucleatum* (MBC: 31.25 μg mL^−1^), compared to the control group, ^F^G_d_ hydrogel, which exhibited no antibacterial activities, silver ions as positive control (see ESI, Fig. S8†). In Fig. 10c and d, the diameters of *Fusobacterium nucleatum* inhibiting loops were approximately 0.57, 1.3, 1.9, 2.67 mm for ^F^G_d_Ag hydrogel 31.5, 62.5, 125, and 250 μg mL^−1^, respectively. Therefore, these findings suggested that ^F^G_d_Ag might be a possible biomaterial for antimicrobial medical applications.

## Conclusion

In summary, the detailed crystallization process of ^F^G_d_ hydrogel was studied using SEM. The results indicated that the micron-scale slice-like structure gradually grew to piece-like and even colorless millimeter-scale granule-like structure while the gel transformed to single crystal, which is, to the best of our knowledge, the first free guanosine-based nucleoside crystal formed from a hydrogel. The single crystal X-ray diffraction analysis indicated that ^F^G_d_ adopted an *anti*-conformation with an N-type (3′-*endo*) sugar puckering and 5′-OH at ap position, formed mimic reverses Watson–Crick base pairs with an intermolecular hydrogen-bond (N10H–O11), and had nine hydrogen bonds infinitely repeated in the entire assembly to form the 3D multilayered supramolecular structure in the solid state. These hydrogen bonds were also further investigated using VT NMR in solution state, and the results were consistent with the solid state. Then, Ag^+^ was introduced to block its crystallization and form hydrogel with long lifetime stability (>6 months). Rheological measurements revealed that ^F^G_d_Ag gel has a higher storage modulus *G*′ than loss modulus *G*′′ over the entire applied frequency range (solid-like behavior). SEM and AFM studies demonstrated that flexible fibers with a diameter of approximately 20–30 nm, height measurements of approximately 7 nm, and length of several micrometers were formed spontaneously during the formation of ^F^G_d_Ag gel whereas ^F^G_d_ formed a slice structure. Possible silver-mediated base pair motifs were suggested using PXRD, CD NMR, UV, and MS. Finally, ^F^G_d_Ag hydrogel exhibited low toxicity for NOK-SI cell and good antibacterial activities for *Fusobacterium nucleatum in vitro*, whereas ^F^G_d_ exhibited no antibacterial activity. The above results suggested that ^F^G_d_Ag might be useful for future applications in the field of antimicrobial medicines or drug delivery.

## Conflicts of interest

The authors declare no conflict of interest.

## Supplementary Material

RA-008-C8RA02500B-s001

RA-008-C8RA02500B-s002
