# Supplementary material for: Silver ions blocking crystallization of guanosine-based hydrogel for potential antimicrobial applications
Source: RSC Adv. 2018 Apr 27;8(28):15842–52. doi: 10.1039/c8ra02500b (PMC9080096; doi:10.1039/c8ra02500b)
Supplement: RA-008-C8RA02500B-s001 [file RA-008-C8RA02500B-s001.pdf]

Electronic Supplementary Material (ESI) for RSC Advances.
This journal is © The Royal Society of Chemistry 2018

Supplementary Information for:

Silver ions blocking crystallization of guanosine-based hydrogel for potential antimicrobial applications †

Hui Feng[†ab], Yuqi Du[†a], Fan Tang[a], Ning Ji[a], Xuefeng Zhao[*a], Hang Zhao[*a], Qianming Chen[a]

[a]*State Key Laboratory of Oral Diseases, West China Hospital of Stomatology, Sichuan University, Chengdu, Sichuan 610065610041, P. R. China. E-mail: zhao.zxf@gmail.com; zhaohangahy@scu.edu.cn.*

[b]*XiangYa Stomatological Hospital, Central South University, Changsha, Hunan 410000, P. R. China*

†Electronic supplementary information (ESI) available: Experimental section and additional figures. See DOI: xxx

[†]These authors contributed equally to this work.

Table of Contents

1. Experimental section…………………………………………………………......3

2. Fig. S1 The possible self-assembling process, SEM and AFM images of $^{F}G_{d}$ hydrogel………………………….………..……..…………..……..…………5

3. Fig. S2 VT NMR spectra of $^{F}G_{d}$….…..………..…………..……………………..……6

4. Fig. S3 ^{1}H-^{1}H NOE spectra of $^{F}G_{d}$………...……………………………….………....7

5. Fig.S4 CD spectroscopy of $^{F}G_{d}$Ag hydrogel.……………………….……….…..8

6. Fig. S5 PXRD patterns of lyophilized of $^{F}G_{d}$Ag and $^{F}G_{d}$ hydrogel………………..9

7. Fig. S6, Table S1.^{1}H NMR spectra of $^{F}G_{d}$ and $^{F}G_{d}$Ag..……………..…………….10

8. Fig. S7 ESI-MS analysis of $^{F}G_{d}$Ag………………………...…….…………….....11

9. Fig. S8. Antimicrobial efficiencies of silver ions *in vitro*………..……………….….12

Experimental Section

All the chemicals were commercially available. The solvents and reagents were analytically pure. NMR spectra were recorded using an AV II (Bruker, Germany) spectrometer at 400 MHz and 600 MHz, and the δ values in ppm were relative to Me_4Si as the internal standard. High-resolution mass spectra were measured using a mass analyzer (Q-TOF, Bruker, Germany). The UV absorption spectra were recorded using a DU-800 spectrophotometer (Beckman, US).

General procedure for hydrogel preparation

A certain amount of nucleosides was suspended in 200 μL of solution of 0.2M KCl or in different concentrations of $AgNO_3$ solution in a glass vial. The mixture was heated until it became a clear solution. Subsequently, the solution was gradually allowed to cool to room temperature. The sample was subjected to a "tube-inversion test." The compound was said to be a hydrogel if no sample flow was observed upon inversion of the tube at room temperature.

NMR spectra

NMR spectra were obtained using AV II spectrometers (Bruker, Germany) at 600 MHz for NOE and VT-NMR, where the values were in ppm relative to Me_4Si as the internal standard. The compounds were dissolved in d_6-DMSO (10 mg/mL).

SEM imaging

SEM was performed using a high-resolution INSPECT F50. The hydrogels were prepared in a sample tube (0.7 w/v %) and were thereafter frozen. The frozen samples were evaporated using a vacuum pump. Prior to examination, the xerogel was attached to the silica wafer and coated with a thin layer of gold.

AFM imaging

AFM measurements were performed in tapping mode at the amplitude setpoint of 1 V using SPI4000 (Seiko Instruments, Chiba, Japan). Soft silicon cantilevers were chosen (SI-DF2000, K-A102001604, Japan) with the spring constant of 5 N/m. Clean mica substrates (1×1 cm^2) were prepared and 5 μL of the samples were deposited onto a freshly cleaved mica surface. The mica surface with the adsorbed samples was

thereafter dried in air and imaged immediately.

Single crystal analyst

Single crystals were obtained by allowing the hydrogel to grow slowly. The crystal data were collected using Bruker Apex II. Crystallographic data were deposited with the Cambridge Crystallographic Data Centre (CCDC reference number: 1588934).

***In vitro* cytotoxicity assay**

The cytotoxicity of the components of $^{F}G_{d}$ and $^{F}G_{d}Ag$ hydrogel was analyzed with normal oral keratinocyte cells (NOK-SI). The cells were seeded in 96-well plates with a density of 10000 cells per well and incubated overnight at 37 ℃. Subsequently, 50 μL of phosphate-buffered saline as a control group and 50 μL of $^{F}G_{d}$ and $^{F}G_{d}Ag$ hydrogel at different concentrations (0, 2, 4, and 8 mg/mL) were added to the cell media and thereafter incubated with the cells for 24 h at 37 ℃. Subsequently, a cell counting Kit-8 (CCK8) assay was carried out to determine the cell viability.

Antimicrobial Activities of the $^{F}G_{d}$ and $^{F}G_{d}Ag$ hydrogels

The antimicrobial activity of the $^{F}G_{d}$ and $^{F}G_{d}Ag$ hydrogels were tested using DENSIMAT method. The bacteria strains used in this work were *fusobacterium nucleatum (Fn)* and *porphyromonas gingivalis (Pg)*. The inhibition growth diameter and minimal bactericidal concentrations (MBCs) of $^{F}G_{d}$ and $^{F}G_{d}Ag$ hydrogels were determined and the process was carried out in triplicate. The antibacterial activities were examined after incubation at 37 ℃ for 48 h under anaaerobic conditions.

Date analysis

The results from replicates were presented as mean ± standard deviation (SD) and the statistical comparisons between different groups were subjected to one-factor analysis of variance. Significant differences were considered as a P value < 0.05 in all experiments.

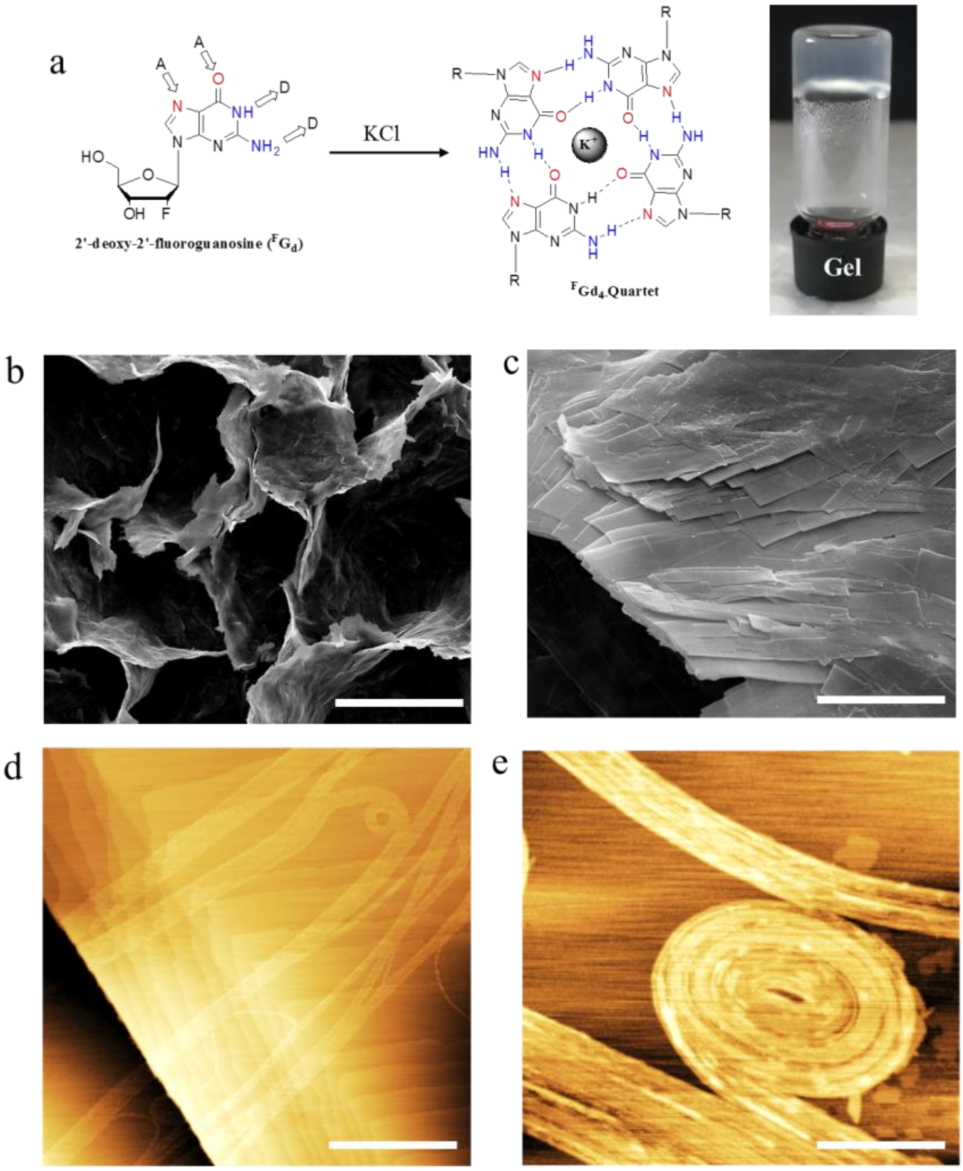

Fig. S1 a) The possible self-assembling process of $^{F}G_d$ hydrogel in the presence of 0.2 M KCl solution. b-c) SEM images of $^{F}G_d$ hydrogel at concentrations of 1.4 % in the presence of 0.01 M KCl solution. d-e) AFM images of $^{F}G_d$ hydrogel at concentrations of 1.4 % in the presence of 0.01 M KCl solution. Scale bars: 100 μm (b), 10 μm (c), 1.0 μm (d), 200 nm (e).

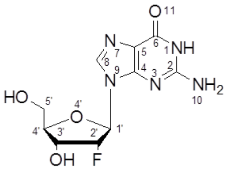

$^{F}G_d$

Temperature	N1H	C8H	N10H	C1'H	3'OH	5'OH
298K	10.73	7.99	6.60	6.02	5.69	5.15
338K	10.73	7.99	6.46	6.02	5.53	4.97
Δδ (ppm)	0	0	0.14	0	0.16	0.18

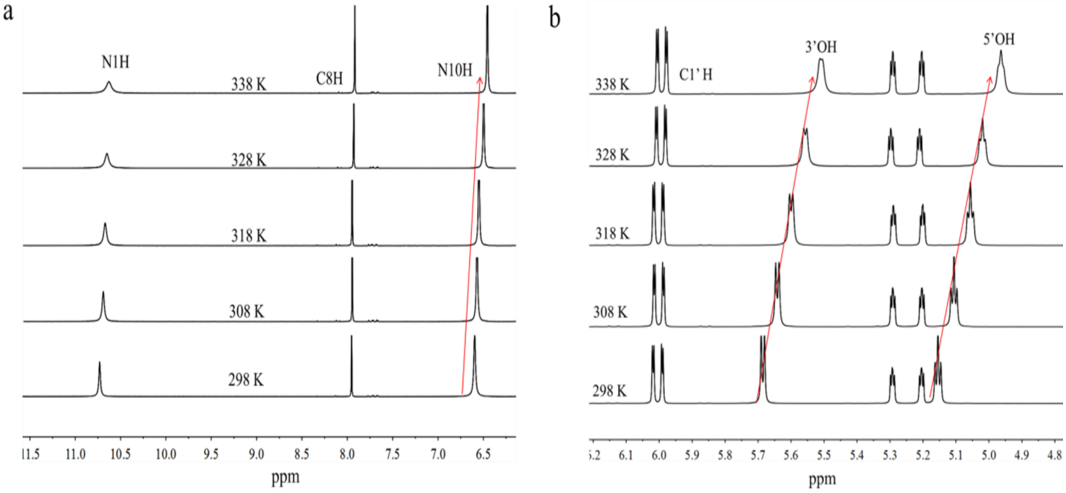

Figure S2. a, b) VT ^{1}H NMR spectra of $^{F}G_d$ (0.1 M / L) recorded from 298 K to 338 K.

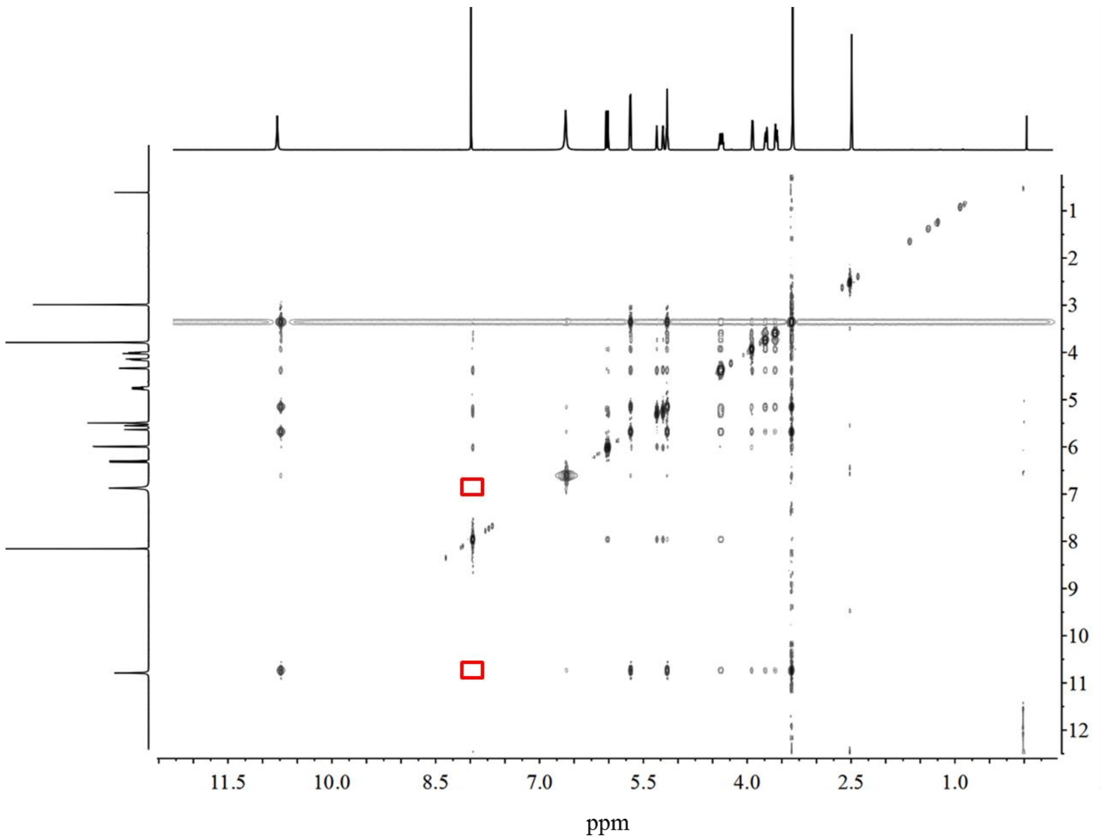

Figure S3. ^{1}H-^{1}H NOE spectra of $^{F}G_{d}$ (0.1 M / L) at 298 K.

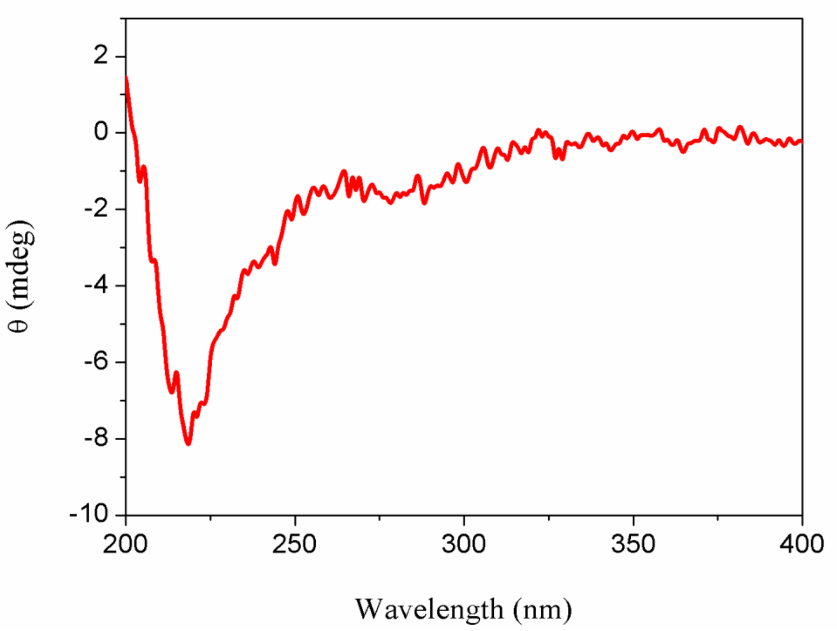

Figure S4. The self-assembly of $^{F}G_{d}Ag$ hydrogel was confirmed by CD spectroscopy.

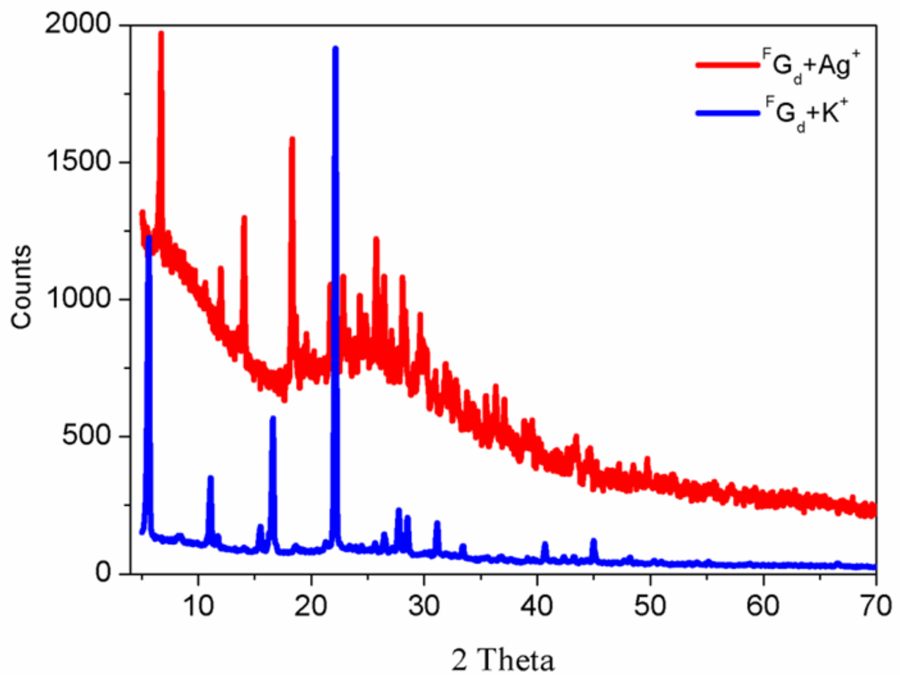

Figure S5. PXRD patterns of lyophilized of $^{F}G_{d}$Ag hydrogel and crystal of $^{F}G_{d}$ hydrogel in the presence of K^{+}.

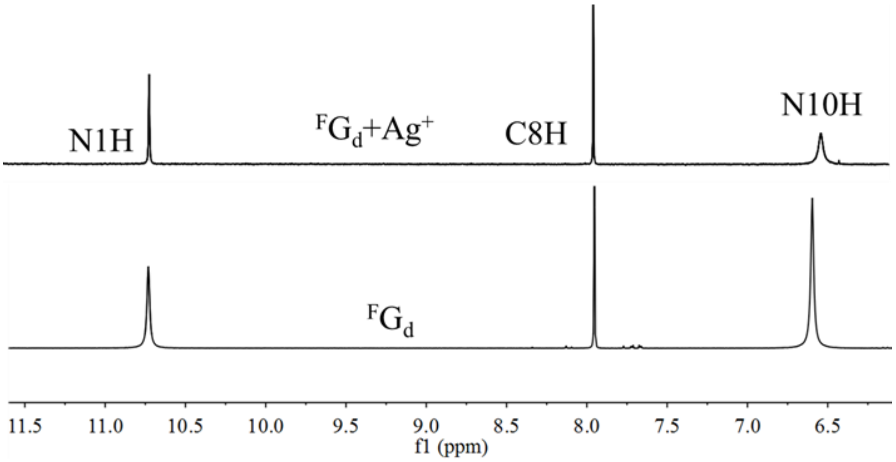

Figure S6. ^{1}H NMR spectra of $^{F}G_{d}$ and $^{F}G_{d}Ag$ (0.1 M / L) recorded from 298 K.

Table S1. VT ^{1}H NMR spectra of $^{F}G_{d}Ag$ recorded from 298 K to 338 K.

Temperature	N1H	C8H	N10H	C1'H	3'OH	5'OH
298K	10.84	8.06	6.65	6.05	5.69	5.16
338K	10.64	8.06	6.47	6.05	5.53	4.98
Δδ (ppm)	0.20	0	0.18	0	0.16	0.18

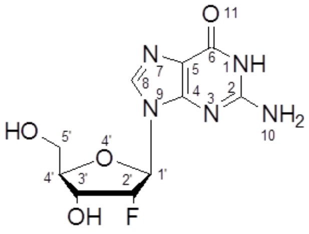

$^{F}G_{d}$

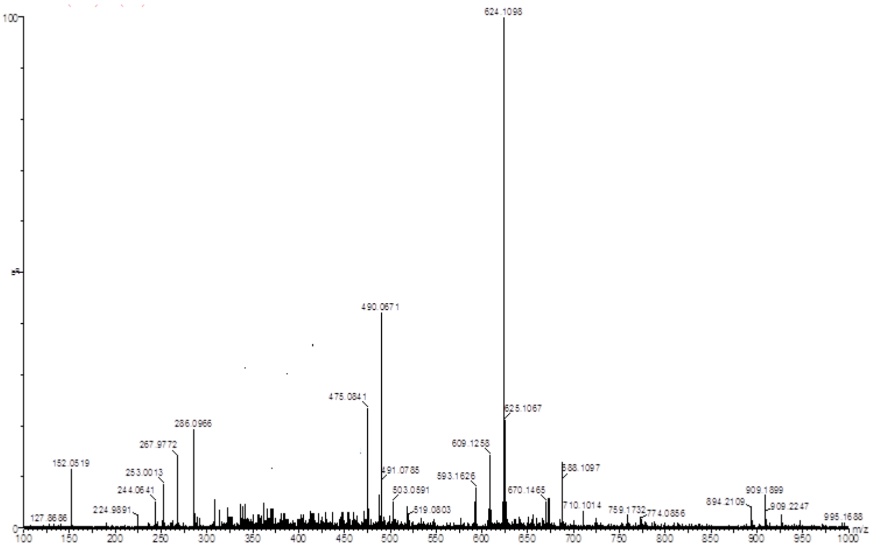

Figure S7. Positive mode ESI-MS analysis ${}^{F}G_{d}$ silver complexes of 1:1 and 1:2 peaks at 475.1 , 624.1.

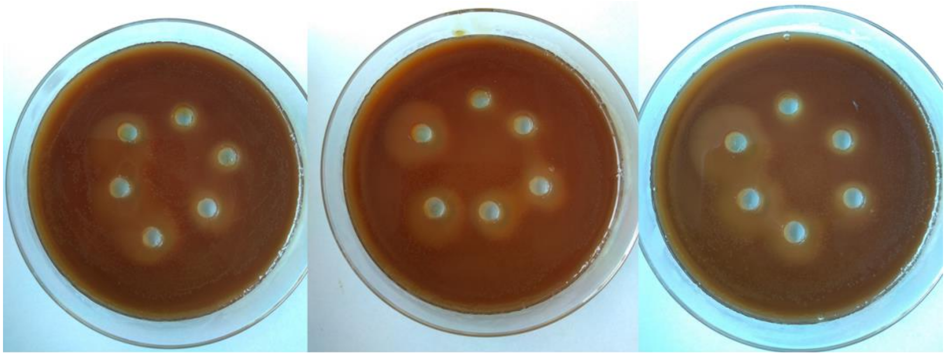

Silver ions Concentration (μg/ml)	Fusobacterium nucleatum (Fn)		
	Inhibition Growth Diameter (mm)		
	1	2	3
**250**	24	21	20
**125**	20	17	18
**62.5**	15	14	16
**31.5**	13	12	12
**15.5**	8	9	10
**0**	0	0	0

Figure S8. Demonstration of excellent antimicrobial efficiencies of silver ions *in vitro*. In the sterilization experiment, the concentrations of silver ions were 0 $\mu g \bullet ml^{-1}$; 15.5 $\mu g \bullet ml^{-1}$; 31.5 $\mu g \bullet ml^{-1}$; 62.5 $\mu g \bullet ml^{-1}$; 125 $\mu g \bullet ml^{-1}$ and 250 $\mu g \bullet ml^{-1}$, respectively. The dates represent the growth inhibition diameter (in mm) of each sample in three independent experiments.
